# 2018 ISCB Overton Prize awarded to Cole Trapnell

**DOI:** 10.1371/journal.pcbi.1006163

**Published:** 2018-06-07

**Authors:** Christiana N. Fogg, Diane E. Kovats, Ron Shamir

**Affiliations:** 1 Freelance Science Writer, Kensington, Maryland, United States of America; 2 International Society for Computational Biology, Bethesda, Maryland, United States of America; 3 Blavatnik School of Computer Science, Tel Aviv University, Tel Aviv, Israel

**Figure:**
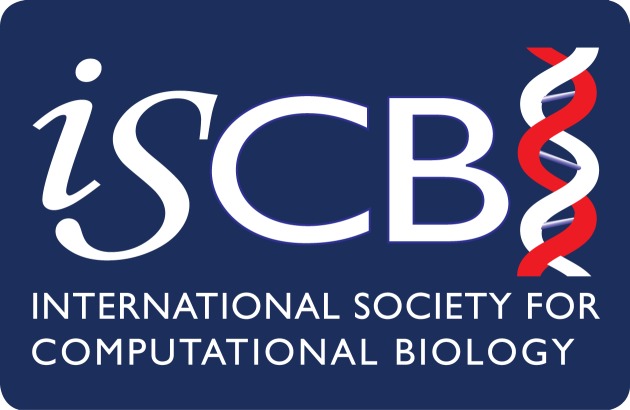


Each year, the International Society for Computational Biology (ISCB) recognizes the achievements of an early- to mid-career scientist with the Overton Prize. This prize honors the untimely death of Dr. G. Christian Overton, a respected computational biologist and founding ISCB Board member. The Overton Prize recognizes independent investigators who are in the early to middle phases of their careers and are selected because of their significant contributions to computational biology through research, teaching, and service.

ISCB is pleased to recognize Dr. Cole Trapnell, Assistant Professor of Genome Sciences at the University of Washington, as the 2018 winner of the Overton Prize. Trapnell will be presenting a keynote presentation at the 2018 International Conference on Intelligent Systems for Molecular Biology in Chicago, Illinois, being held from July 6–10, 2018.

## Cole Trapnell: Building bridges to the lab bench

**Fig 1:**
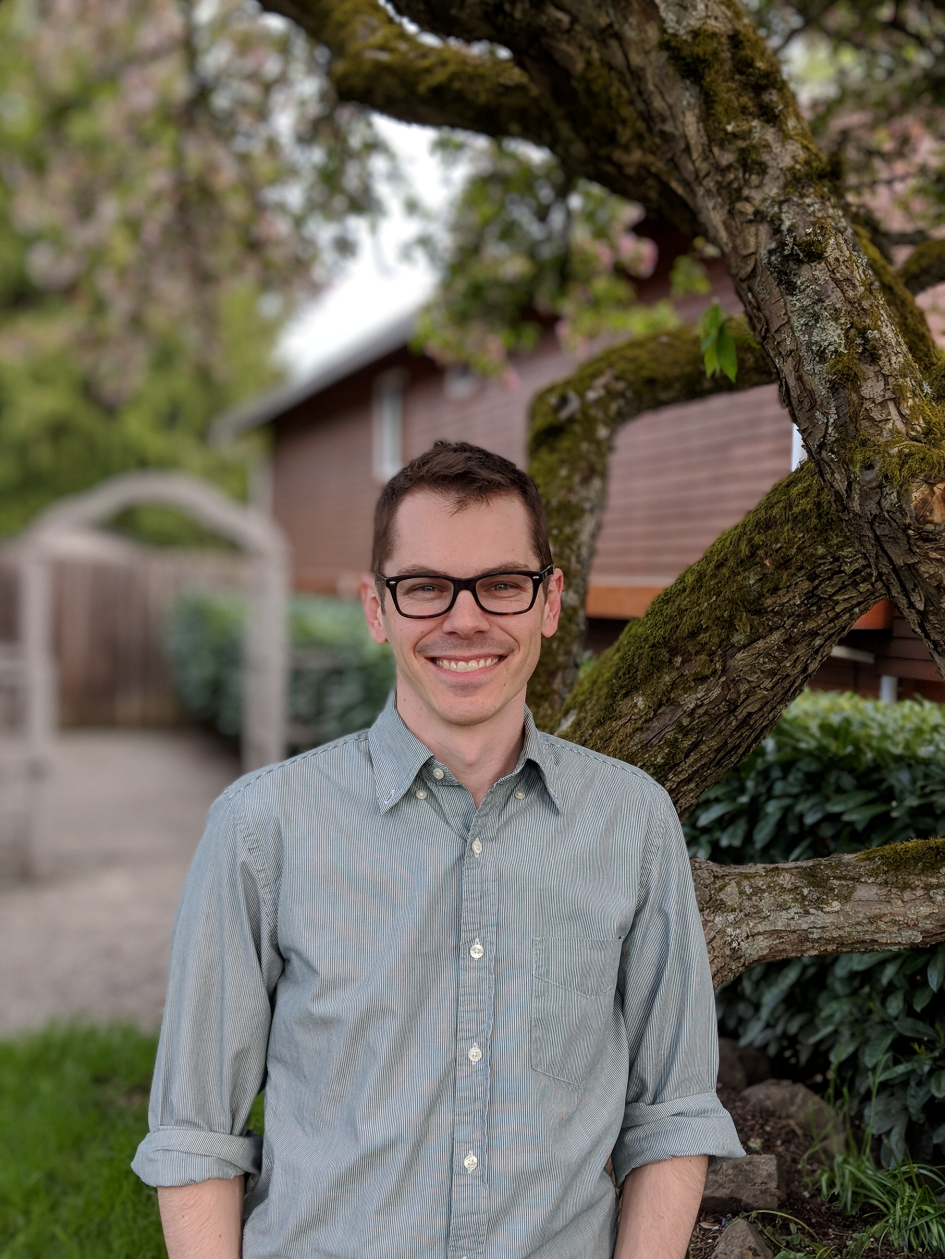
Cole Trapnell.

Cole Trapnell’s (Fig 1) earliest interest in science began at home. He was born in Cheverly, Maryland, and spent his childhood living in College Park, right near the University of Maryland. His father, Bruce Trapnell, is a physician scientist, and Cole has fond memories of accompanying his father to the lab. Beyond the hands-on experiences of doing restriction digests with his dad as a young child, Trapnell most appreciates how his father encouraged him to think scientifically. He recalled, “One time we were playing a board game, and I remarked that because the last dice roll was a six, the next one wouldn’t be. My dad decided to correct my thinking, so the next thing I knew, we were flipping a penny 1,000 times to estimate the probability distribution of getting heads versus tails. I still have the plot that we drew by hand on 1-mm graph paper”.

Trapnell was first interested in physics and abstract mathematics and was drawn to how these fields tackled complex ideas in terms of “first principles.” He began learning programming as a high school student and worked as a student engineer on a robotics project for the United States Army. Trapnell honed his coding skills as an undergraduate by working for a startup that developed software for the areas of retail stock, futures, and foreign currency trading, and he learned how to develop tools that could do complex calculations with large amounts of data in real time. He completed a dual Bachelor of Science degree in computer science and mathematics at the University of Maryland, College Park in 2005 and then began his PhD in computer science there as well. Trapnell thought he would work on problems in supercomputing, but then he took Steven Salzberg’s class on bioinformatics. This brought his attention to the emergence of “next-generation” sequencing technology, and he realized the potential for high-throughput computing to handle this sequence data.

Trapnell’s PhD research focused on sequence alignment, and he adapted the Bowtie algorithm developed by Ben Langmead into a program called TopHat that could handle transcriptomic data. During this time, Trapnell moved to the University of California, Berkeley, where his wife was pursuing her PhD in mathematics, and he started working with Lior Pachter, who became his co-advisor with Salzberg at UMD. As Trapnell developed TopHat and the companion tool, Cufflinks, he tested them with data sets from Barbara Wold’s lab, and he began to develop an appreciation for biological questions, especially in gene regulation. Trapnell was drawn to doing bench research, and his labmate Rob Bradley encouraged him to take that leap. He recalled, “Rob Bradley convinced me that to become a really good biologist, I should learn to do experiments. Rob, who trained as a biophysicist, had gone off to do a postdoc at the bench. I followed suit and joined John Rinn’s lab (at Harvard University), where I worked to both do experiments and analyze them myself.” Trapnell’s time in Rinn’s lab not only helped him get his hands dirty doing bench research but gave him the unique perspective of working under a scientist who pioneered the field of long noncoding RNAs.

Trapnell’s postdoctoral training opened his eyes to the realities of experimental biology, and he acknowledges that these experiences have made him a better computational biologist. While Cufflinks could help him predict which individual splice isoforms may be elevated under certain disease conditions, he came to realize how hard it can be to validate these observations at the lab bench: a specific antibody may not exist for a western blot, or technical difficulties may make it difficult to knock down a gene isoform in a particular model system. Trapnell had to adjust to the different culture associated with working in a wet lab. He recounted, “Computational people are often mystified and frustrated by how often their experiments fail. I like to tell them a story of my own frustration: A little while after starting my wet lab postdoc training, I was complaining to my labmate, Dave Hendrickson, that my experiments were constantly failing. He asked me how long I’d been at it, and I told him about six months. He said, ‘Well, give it another six months.’ I thought he meant I would get better at doing experiments, but what he actually said next was, ‘It’ll hurt less when they don’t work.’ This was a tremendously eye-opening thing for me, because he was trying to tell me that being an effective experimentalist means anticipating failure, planning for it, designing controls that can detect it, and parallelizing work within projects so that you can make progress in one direction even when you’re stuck in another. There are similar cultural differences that experimentalists encounter when learning to program.” As a principle investigator (PI), Trapnell is supportive of students and trainees that want to gain both experimental and computational experience, but he wants to them to learn to understand the culture of these two realms and not just acquire the necessary skills to do experiments or develop algorithms.

Throughout his training, Trapnell has valued the guidance of his mentors. His current lab is positioned between the labs of Stan Fields and Bob Waterson, both leaders in the field of genomics, and they have been invaluable advisors to Trapnell. He said, “Despite their fame and their busy lives, both go way out of their way to advise me on how to bring my research and lab to its potential.” All of his mentors have inspired Trapnell to build a lab culture that encourages open, inspiring and rigorous science. As he established his own lab at the University of Washington, he has started to think differently as a PI and said, “I am continually faced with the question: What do I think is the most important scientific contribution I can make?” Shifting his mindset has been a challenge, but he is still broadly interested in gene regulation, especially gaining a more quantitative understanding of the epigenome. Trapnell considers the advances in single-cell measurements as critical to quantifying aspects of gene regulation, and his team is developing tools for single-cell measurements of gene expression, chromatin accessibility, and other features of the molecular state of the genome. Much of this work is in collaboration with Jay Shendure, whose lab specializes in molecular biotechnology development. Trapnell is keen on this collaboration: “Jay and I have very different approaches but share a common goal to transform our understanding of development and disease using single-cell technologies. Our collaboration has been fantastically productive and fun so far, and there’s a lot more to come”.

Trapnell is deeply honored to be selected for the Overton Prize and said, “I feel strongly that my success is at least as much a product of my being in the right place at the right time with the right collaborators as from any choices I made. I have been repeatedly given great opportunities and I’ve tried to make the best use of them, but I would have gotten nowhere if not for the generous help and creativity of a long list of mentors, collaborators, and colleagues”.

